# Deep Edge-Based Fault Detection for Solar Panels

**DOI:** 10.3390/s24165348

**Published:** 2024-08-19

**Authors:** Haoyu Ling, Manlu Liu, Yi Fang

**Affiliations:** 1 School of Information Engineering, Southwest University of Science and Technology, Mianyang 621000, China; 2 School of Information Science and Technology, University of Science and Technology of China, Hefei 230026, China

**Keywords:** solar panels, thermography, fault detection, edge detection

## Abstract

Solar panels may suffer from faults, which could yield high temperature and significantly degrade their power generation. To detect faults of solar panels in large photovoltaic plants, drones with infrared cameras have been implemented. Drones may capture a huge number of infrared images. It is not realistic to manually analyze such a huge number of infrared images. To solve this problem, we develop a Deep Edge-Based Fault Detection (DEBFD) method, which applies convolutional neural networks (CNNs) for edge detection and object detection according to the captured infrared images. Particularly, a machine learning-based contour filter is designed to eliminate incorrect background contours. Then faults of solar panels are detected. Based on these fault detection results, solar panels can be classified into two classes, i.e., normal and faulty ones (i.e., macro ones). We collected 2060 images in multiple scenes and achieved a high macro F1 score. Our method achieved a frame rate of 28 fps over infrared images of solar panels on an NVIDIA GeForce RTX 2080 Ti GPU.

## 1. Introduction

According to the latest report [1], renewable energy sources such as solar and wind systems will meet 88% of the global energy demand by 2050. As an alternative and a critical complement to thermal power, solar energy plays a crucial role in supplying more and more pollution-free electric power. At the same time, with the rapid development of the photovoltaic industry, the daily maintenance and operations of photovoltaic power stations are facing significant challenges, particularly the faults of solar panels. Studies reveal that the annual solar power loss due to various faults, such as snail trails, discoloration, and corrosion [2], is 18.9% [3]. Patrol is a crucial part of photovoltaic power stations’ safety production, which can help ensure that all electrical systems and wires meet legal safety standards.

As solar panels are generally laid on hillsides, plains, swamps, lakes, rooftops, and other inaccessible areas, one patrol task may take several weeks by several patrollers, which is challenging to meet the demand in terms of efficiency and frequency. With the wide applications of Unmanned Aerial Vehicles (UAVs) in surveillance [4], wildlife protection [5], and infrastructural inspection [6], more and more photovoltaic power stations are using drones for inspection. UAVs can capture video streams in just a few hours and surpass geographical limitations. Due to the characteristics of low cost, high flexibility, and simple operations, UAVs are very popular in equipment inspection. Infrared (IR) imaging allows fast and straightforward identification of overheated solar panels, also known as thermography. The low signal-to-noise ratio of thermography can better highlight faults than normal visible images.

Among the major faults of solar panels, dotted hotspots are usually formed due to shading or breakage, while circuits cause rectangular hotspots. Although UAVs can free patrollers from complicated tasks, the analysis of infrared images has always troubled them. Inspectors have to mark the fault location from a huge number of infrared images, which is time-consuming and depends on the staff’s experience. The purpose of this paper is to develop an automatic fault detection algorithm for infrared images of solar panels to reduce the inspection cost of the photovoltaic industry.

Automatic fault detection of photovoltaic panels can be decomposed into two sub-tasks: location and classification, i.e., where and which category faults of a solar panel are. It is a challenging task and has caught more and more attention. There are three types of images, IRT (infrared thermography) [7,8,9,10,11], EL (electroluminescence) [12,13,14,15,16], and PL (photoluminescence) [17,18]. Traditionally, methods based on hand-crafted features [9,11] have been used to determine the location of faults. After obtaining the positions of faults, adopting SVMs (support vector machines) to classify faults is a popular choice of traditional methods [11]. Recent years have witnessed significant progress in fault detection using deep CNNs (convolutional neural networks). Due to the ability of deep learning to express multi-level features, CNN-based methods surpass traditional methods in tasks, such as image classification, target detection, and semantic segmentation. With the rapid development of deep learning in different tasks, many methods have shined light on photovoltaic fault detection. Early methods [8,13,15,17] only solved classification tasks, using CNNs, such as VGG19, to learn deep abstract data representations. Later object detection methods [7,14,16] can output the location and category of faults in an end-to-end manner, e.g., Faster RCNN [19] and YOLO series [20,21,22,23,24]. Few semantic segmentation methods [12] demonstrated their feasibility in a small number of faulty samples. Nevertheless, the above techniques are prevented from having practical applications due to the following reasons: First, EL images need to be acquired by professional instruments, which is only applicable in laboratories. Second, traditional descriptor-based approaches require complicated parameter settings and entirely rely on experts’ experience. Third, the diversity of drone flight attitudes and altitudes may cause fault detection algorithms to fail in some cases. Finally, class imbalance is the most common problem in photovoltaic (PV) fault detection (the fault rate is normally lower than 4%), showing biased results. At the same time, automatic fault detection of solar panels has become a vital issue for efficient daily inspections.

**Figure 1:**
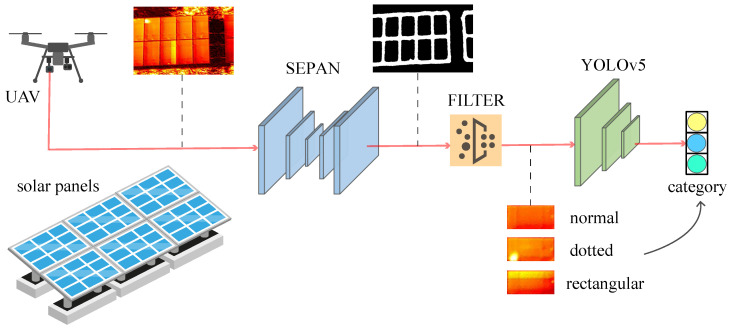
The pipeline of DEBFD includes three steps, edge detection (SEPAN), contour filter, and classification (YOLOv5). The task of DEBFD is to locate and identify normal, dotted, rectangular solar panels in infrared images.

To address the above challenges, we pay attention to edge detection, a fundamental computer vision problem. Recent literature has proved that CNNs have greatly surpassed traditional methods in edge detection tasks on public datasets, and modern CNNs can learn rich, hierarchical edge representations. Why not use CNN-based edge detection to locate solar panels? On the one hand, an excellent edge detector can be trained on the BSDS500 [25] dataset, which inspires us to collect datasets at a small cost. On the other hand, infrared images are only affected by temperature so that the edges of solar panels are clearly visible under sufficient sunlight. The edge features are still highly adaptable to locate solar panels for UAV aerial photography with scale and rotation ambiguity characteristics. Based on the above analysis, we construct a general framework for automatic fault detection of solar panels. As shown in Figure 1, the framework consists of three parts: edge detection, contour filter, and classification. The goal is to find the location of faulty solar panels from infrared images. Edge detection finds the positions of all solar panels in the image, which is performed by finding the contour of the mask image of the edge. Then the contour filter algorithm will delete the background contour to eliminate the clutter in the background. Lastly, solar panels are classified into normal, dotted faulty, and rectangular faulty. Actually, both dotted faulty and rectangular faulty solar panels compose faulty panels, which should be handled. The main contributions of this paper are listed below.

1. We develop a novel fault detection pipeline of solar panels, which consists of three steps: (a) edge detection, (b) contour filter, and (c) classification.
2. We adapt existing CNNs for fault detection of solar panels. To the best of our knowledge, ours is the first CNN-based edge detection approach in this task. The proposed bottom–up self-attention structure leads to more detailed edge location information.
3. We collect and annotate 1200 images in different photovoltaic power plants (desert, mountain, roof, water, woodland) and achieve a high macro F1 score in 860 testing images.

## 2. Related Work

### 2.1. Edge Detection

As one of the fundamental image processing tasks, edge detection has a wealth of research literature. Early traditional methods used operators such as Sobel, Prewitt [26], and Canny [27] to pay attention to the gradient changes in local intensity and brightness in the image. The weakness of traditional methods lies in the fact that local noise is fatal to the gradient information so that they can hardly be applied in practical applications. Later, machine learning-based methods become the mainstream of edge detection. Researchers construct feature descriptors by combining intensity, color, and gradient and then use complex learning paradigms to obtain edge intensity, such as in [28,29,30]. Learning-based methods usually yield good results in simple scenarios, but may suffer from a lack of effective acceptable edge representation. With the rise of CNN, deep learning-based edge detectors have been proposed recently. Xie et al. [31] proposed a fast and accurate edge detector, Holistically Nested Edge Detection (HED), which concatenates side outputs of different scales and uses 1 × 1 convolution to fuse them. Wang et al. [32] then used subpixel convolution, instead of bilinear interpolation or deconvolution, which is beneficial for accurate edge localization. Xu et al. [33] first introduced Attention-Gated Conditional Random Fields (AG-CRFs) and considered attention variables as gates, resulting in rich and complementary feature expressions. Later, Liu et al. [34] exploited all the convolutional layers of each stage of the primary network to extract rich features. Deng et al. [35] proposed a new loss function to generate fine edges, which alleviated the problem of class imbalance. He et al. [36] developed a Bi-Directional Cascaded Network to perform a different supervision on multi-scale features, superior to human perception on the BSDS500 dataset. Su et al. [37] designed a novel pixel difference convolution inspired by traditional edge detection operators, which can achieve more than 100 FPS (frames per second) on an NVIDIA RTX 2080Ti GPU.

### 2.2. Fault Detection of PV Panels

The purpose of fault detection of solar panels is to implement image processing technology to reduce the burdens of operation and maintenance. Existing methods can be roughly divided into three categories: (1) statistical features, (2) machine vision, and (3) deep learning. Dotenco et al. [9] statistically modeled temperature to locate solar panels and identify overheated areas such as Gaussian distribution, median, and histogram. Machine learning methods focus on the texture features of the image after filtering. For example [10,11], all improve the Canny operator to obtain the diverse expression of the edge. Deep learning methods achieve promising performance, and find many large-scale applications. Li et al. [17] proposed an unsupervised clustering method for the potential embedding of data features to overcome missing labels caused by sparse fault samples in industrial situations. Akram et al. [8] transferred learning from the EL dataset and increased the average accuracy rate from 98.67% to 99.23%. Su [16] embedded the channel and spatial position attention mechanism in Faster RCNN’s RPN (Region Proposal Network) to extract more refined defect region proposals. On EL images, Rahman et al. [12] defined a hybrid loss function (dice loss combined with focal loss) to train a multi-attention UNet network to help overcome the problem of data imbalance. Although these methods have used CNNs to promote the development of the field to some extent, they have not fully considered the characteristics of photovoltaic images taken by drones, namely, scale and rotation ambiguity. To resolve this issue, we will propose an edge-based framework for complete location and classification.

## 3. Materials and Methods

This section presents our Deep Edge-Based Fault Detection (DEBFD) method for solar panels, including edge detection, contour filter, and classification. For the captured infrared images, the pseudocolor mode is fulgurite.

### 3.1. Edge Detection

**Figure 2:**
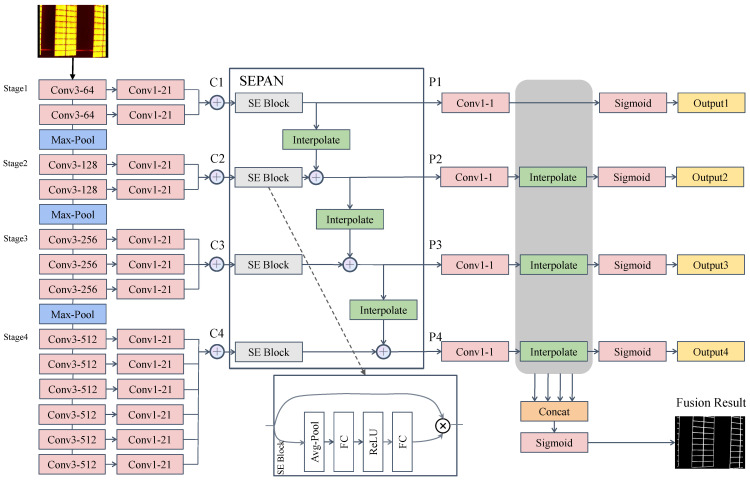
SEPAN architecture. Conv1-21 means convolution with 1 × 1 kernel size and 21 channels.

Our edge detection network, called SEPAN, uses a backbone modified by VGG16 [38] and a neck with a squeeze-and-excitation path aggregation structure. As shown in Figure 2, the input of SEPAN is an infrared image, and its output is an edge map, which represents the probability for each pixel to belong to edges.

#### 3.1.1. Backbone

As in [31,34], we pre-train VGG16 on ImageNet as our backbone. VGG16 has 5 stages and 13 convolutional layers, whose each stage is followed by a pooling layer. From stage 1 to stage 5, the receptive field keeps increasing and contains more and more contextual information. Unfortunately, the receptive field increase is still limited for our task. The experimental results of HED [31] will show that the side-output layer 5 (connected with stage 5) produces relatively low performance. Inspired by this observation, we keep only the first three pool layers and merge stages 4 and 5. Like RCF [34], the convolutional layer of each stage is connected to a 1 × 1 convolutional layer with a channel depth of 21. Then the results of each stage are added element-wise to produce hybrid features.

#### 3.1.2. Squeeze-and-Excitation Path Aggregation Structure

Existing works [39,40,41,42] have obtained high-level semantic features by fusing multi-scale information on object detection and semantic segmentation tasks. Our network design aims to design effective edge multi-scale representations. Top–down and bottom–up structures are two common ways of semantic fusion. Unlike the top–down one, a clean horizontal connection path is established from low to high. Edge detection can be regarded as a special kind of semantic segmentation, and the bottom–up path enhancement is critical for positioning in semantic segmentation. It facilitates high-level features to access low-dimensional positioning information. For more experiment results, please refer to Section 4.3. We also added channel attention to the bottom–up structure, Squeeze-and-Excitation Block (SE Block), which was first proposed by [43]. Specifically, we replaced each lateral vanilla convolution with an SE Block. As shown by the dotted line in Figure 2, the SE Block sequentially generates a set of self-attention weights with the number of channels through the average pooling, ReLU, and fully connected layers, and multiplies it with the original input feature element-wise to generate the result. With the SE Block, the bottom–up structure can better map the channel dependency and access the global information. Therefore, it can better recalibrate the filter outputs, which leads to good edge representation. Our framework accepts multi-scale features $\left\{C_{1},C_{2},C_{3},C_{4}\right\}$ from the backbone and outputs enhanced features $\left\{P_{1},P_{2},P_{3},P_{4}\right\}$ with the same spatial size. From C1 to C4, the spatial size is gradually reduced with a factor of 2. Each output layer adopts a higher-resolution feature map $P_{I}$ and a lower-resolution map $C_{I+1}$ through element-wise addition, generating a new feature map $P_{I+1}$. $C_{I+1}$ goes through an SE Block, and $P_{I}$ is adjusted to the same spatial size as $C_{I+1}$ through bilinear interpolation. Note that $P_{1}$ is directly generated by $C_{1}$. In this structure, an SE Block does not change the number of channels in the feature map.

#### 3.1.3. Loss Function

After obtaining $\left\{P_{1},P_{2},P_{3},P_{4}\right\}$, they are reduced to a single channel map by a 1×1 convolutional layer, which is then interpolated to the original size, followed by a Sigmoid function to generate the output edge maps $\left\{O_{1},O_{2},O_{3},O_{4}\right\}$. We use the following weighted cross-entropy loss:

(1) $$\begin{array}{c} ł\left(X;Y\right)=-\beta \sum_{y_{j}\in Y^{+}}y_{j}\log p_{j}-\alpha \sum_{y_{j}\in Y^{-}}y_{j}\log p_{j} \end{array}$$

(2) $$\begin{array}{cc} \alpha & =\lambda \cdot \frac{\left|Y^{+}\right|}{\left|Y^{+}\right|+\left|Y^{-}\right|}\\ \beta & =\frac{\left|Y^{-}\right|}{\left|Y^{+}\right|+\left|Y^{-}\right|} \end{array}$$

where *X* denotes the training image, and $Y=\left\{y_{j},j=1,\ldots ,\left|Y\right|\right\}$ is the corresponding ground truth pixel set. $Y^{+}$ and $Y^{-}$ denote the set of edge pixels and non-edge pixels in the ground truth, respectively. $p_{j}$ is the predicted value of $y_{j}$. $\left|Y^{+}\right|$ and $\left|Y^{-}\right|$ denote the pixel numbers of an edge pixel set and a non-edge pixel set of the ground truth, respectively. The hyper-parameter λ is designed to balance the importance of edges and non-edges, and is set as λ=1.1 in our experiments. In particular, we concatenated all layers of all scales to generate a fusion result $O_{fuse}$, which is used for evaluation. During training, $\left\{O_{1},O_{2},O_{3},O_{4},O_{fuse}\right\}$ are all involved in the loss function calculation. Thus, the total loss function is defined as follows:

(3) $$\begin{array}{c} L\left(X;Y\right)=l_{O_{fuse}}\left(X;Y\right)+\sum_{i=1}^{4}l_{O_{i}}\left(X;Y\right) \end{array}$$

### 3.2. Contour Filter

**Figure 3:**
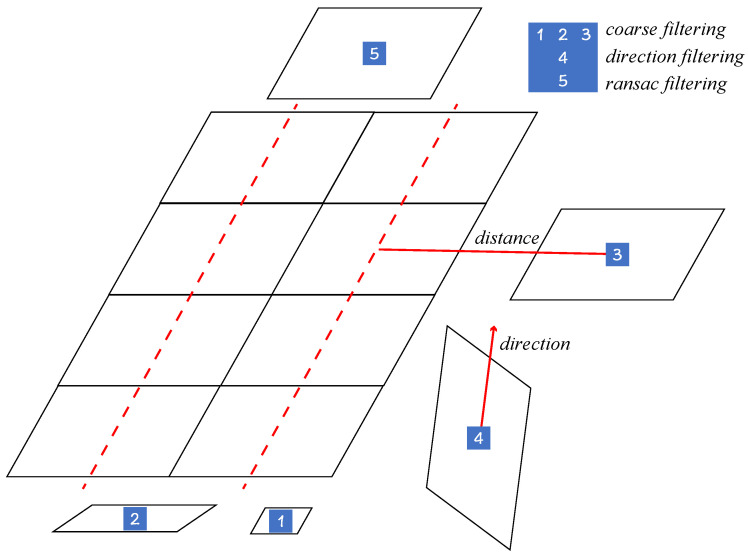
An example of filtering policies. Note that different numbered parallelograms represent different types of filtered background contours.

According to [9,10,11], an effective filtering mechanism can select the correct contour from numerous candidates. Having obtained the binary mask map in Section 2.1, the contour borders are computed by the algorithm in [44]. Then the minimal enclosing parallelogram of each contour is determined to represent a candidate. We define *C* as the set of *N* candidate contours, and a general filtering algorithm can be described as f(C), which is a subset of *C*. The workflow of the proposed filtering strategy can be divided into the following four stages, including minimal enclosing parallelogram, coarse filter, main direction filter, and RANSAC filter. Figure 3 shows parallelograms filtered by our proposed filtering strategies. The specific details will be described later.

#### 3.2.1. Minimal Enclosing Parallelogram

**Figure 4:**
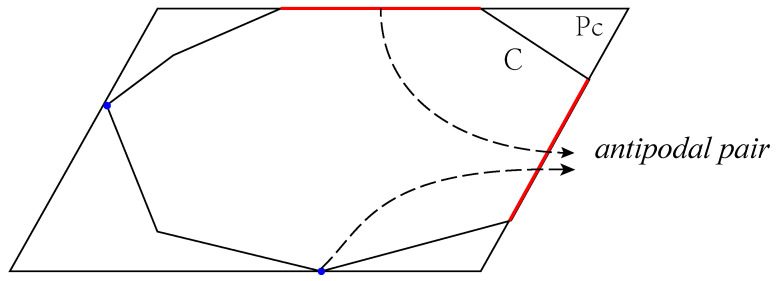
An example of minimal enclosing parallelogram. Note that an antipodal pair includes a red edge and a blue vertex.

As the flight attitude of the UAV is constantly changing in the inspection, the transformation of the solar panel to the imaging plane can be regarded as an affine transformation; i.e., the rectangular solar panel may be mapped into a parallelogram in the infrared image. Given the contour points, we convert each contour point set into the clockwise vertices of a convex polygon *C*. Then *e* and *v* represent an edge and a vertex of *C*, respectively. The pair $\left(e,v\right)$ is called as an antipodal pair of *C* if *v* has the farthest Euclidean distance from *e* among all vertices. For any convex polygon *C*, we denote $P_{c}$ as the minimal enclosing parallelogram among all parallelogram candidates. According to [45], each axis of $P_{c}$ contains an edge of *C*. One example is shown in Figure 4. Therefore, the minimal enclosing parallelogram for the clockwise convex polygon *C* can be calculated as follows:

1. Obtain the list $L=\left\{\left(e_{i},v_{i}\right);i=1,\ldots ,N\right\}$ by the Rotating Calipers algorithm, where vertex $v_{i}$ is the farthest from edge $e_{i}$ among all the vertices of *C*;
2. Sequentially traverse the list *L* and select $\left(e_{j},v_{j}\right)$ and $\left(e_{k},v_{k}\right)$ to determine the unique parallelogram $\left(j\in \left(1,N\right),k\in \left(j+1,N\right)\right)$;
3. Repeat Step 2 until all candidate parallelograms have been processed.

#### 3.2.2. Coarse Filter

We use the coarse filter to identify parallelogram candidates that are essentially impossible to be solar panels. Define the list $P=\left\{p_{i};i=1,\ldots ,N\right\}$, which contains all parallelograms after calculating the minimal enclosing parallelogram of all contours. Considering a priori knowledge about the shape and location of solar panels, the coarse filtering approach consists of the following operations:

1. Filter those parallelograms, e.g., No. 1 in Figure 3, that do not satisfy $t_{area}^{min}<A_{p_{i}}<t_{area}^{max}$, where $A_{p_{i}}$ is the area of $p_{i}$, and $t_{area}^{min}$ and $t_{area}^{max}$ are preset thresholds;
2. Exclude parallelograms, e.g., No. 2 in Figure 3, when the aspect ratio $r_{p_{i}}=w_{p_{i}}/h_{p_{i}}$ is larger than a threshold $t_{ratio}$, where $h_{p_{i}}$ and $w_{p_{i}}$ represent the short side and the long side of $p_{i}$, respectively;
3. Filter those parallelograms, e.g., No. 3 in Figure 3, without candidates nearby, i.e., those parallelograms whose distance to that parallelogram is greater than a threshold $t_{d}$.

Since we do not want that coarse filtering will remove any correct candidate, the above thresholds should be set reliably enough. $t_{area}^{min}$ and $t_{area}^{max}$ are 500 and 30,000, respectively, while the usual area is around 3000, and $t_{ratio}$ is set to 3.0, while the aspect ratio of solar panels is around 1.5. $t_{d}$ is set to $1.5h_{p_{i}}$.

#### 3.2.3. Main Direction Filter

In this study, the orientation $\theta_{p_{i}}$ of the parallelogram is defined as the direction of the long side, where $\theta_{p_{i}}\in \left[0,\pi \right)$. The underlying philosophy of the main direction filtering is that the orientation $\theta_{p_{i}}$ falls within the range of $\left(\theta_{m}-t_{\theta },\theta_{m}+t_{\theta }\right)$, where $\theta_{m}$ represents the main direction of the whole image with the allowed variation value $t_{\theta }$. According to the distribution pattern of solar panels, we find that they are oriented in approximately the same direction in most cases. Based on the above findings, we obtain grids adjacent to each other by equating the angle ranges. Then each parallelogram will vote on these grids. Finally, the grid with the highest score will decide the main direction. The proposed algorithm is summarized as follows:

1. Divide the range of $\theta_{p_{i}}$ equally into $N_{o}$ grids $\left\{G_{i},i=1,\ldots ,N_{o}\right\}$;
2. Update the scores of the grid $G_{i}$, which $p_{i}$ belongs to, and its neighboring grids $\left\{G_{j},j\in \left[i-t_{o},i+t_{o}\right]\right\}$, where $t_{o}$ is the threshold of the voting strategy;
3. The direction of the highest scoring grid is taken as the main direction $\theta_{m}$. Filter parallelograms that do not satisfy the condition $|\theta_{p_{i}}-\theta_{m}|<t_{\theta }$.

**Figure 5:**
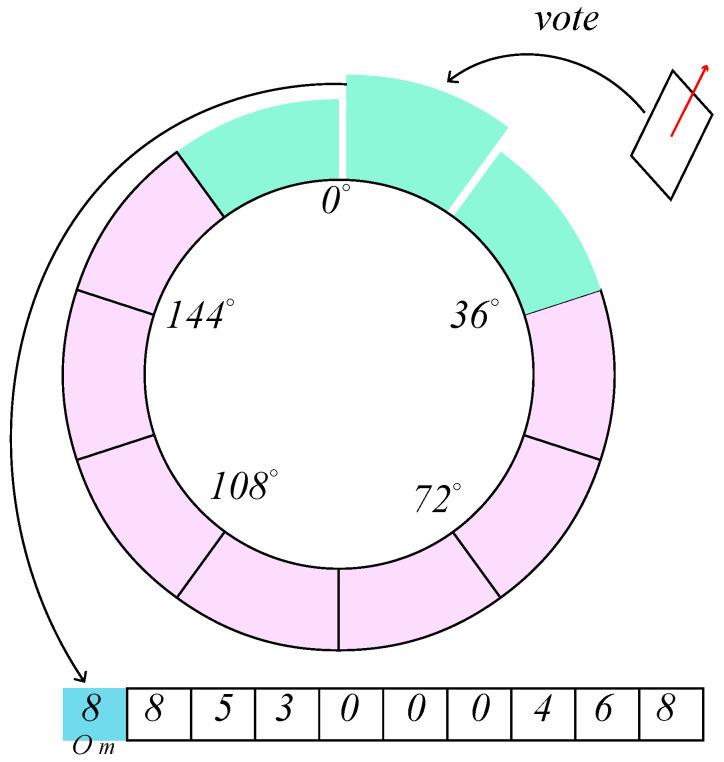
An example of the main direction filtering ($N_{o}=10,t_{o}=1$). Green and purple represent the voted and unvoted grids, respectively. A table of the voting results is also shown at the bottom.

Figure 5 shows an example of $N_{o}=10,t_{o}=1$. For an extreme case that has more than one continuous grid with the highest score, we choose the middle angle of the continuous range as the main direction.

#### 3.2.4. RANSAC Filter

In our study, RANSAC is implemented to eliminate those isolated parallelogram candidates whose centroids are barely in a straight line with the others. Define the set of centroids of all parallelograms as $S=\left\{s_{i},i=1,\ldots ,N\right\}$. Then a simple filter algorithm is given below.

1. Find an optimal straight line *l* using the standard RANSAC algorithm, which has the maximum number of interior points;
2. Remove the interior points of line *l* from set *S* to obtain set $S^{\prime }=\left\{s_{i},i=1,\ldots ,N\;and\;i\notin l\right\}$;
3. Repeat the above steps until S=∅. Then filter the optimal line with the number of interior points less than the threshold $t_{r}$.

**Figure 6:**
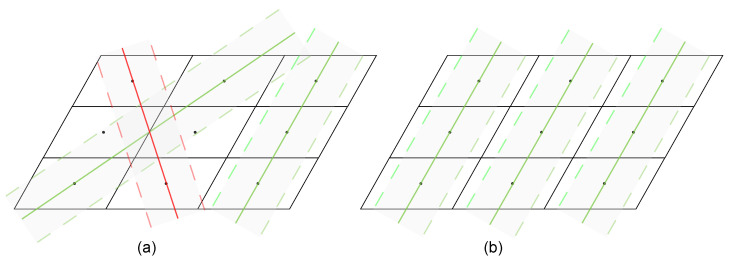
An example of the RANSAC filter when the red line is incorrectly filtered: (**a**) the standard RANSAC algorithm; (**b**) the improved RANSAC algorithm.

It is not practical to apply the above method in realistic scenarios. As shown in Figure 6, randomly selecting two points to construct a straight line can cause a real solar panel to be filtered incorrectly. At the same time, the algorithm relies on the selection of suitable thresholds, and the iterative computation has high time complexity. Taking these factors into account, an improved RANSAC filtering algorithm is constructed as follows:

1. For each centroid $s_{i}$, choose two lines $l_{w}$ and $l_{h}$, where $l_{w}$ and $l_{h}$ represent the direction of the long and short axes of the parallelogram $p_{i}$, respectively;
2. Calculate the number of interior points of 2N lines, where $l_{w}$ and $l_{h}$ have interior point thresholds of $0.3w_{p_{i}}$ and $0.3h_{p_{i}}$;
3. Select one of the optimal straight lines *l*. Since there is an overlap of the interior points of the lines, it is necessary to remove those interior points that are part of the optimal line among the interior points of the non-optimal lines;
4. Repeat Step 3 until all centroids are assigned to an optimal line, filtering out the optimal lines whose number of interior points is less than the threshold $t_{r}$.

The improved RANSAC algorithm only needs to compute the interior points of 2N lines (with the time complexity O(N)) and can adaptively adjust the threshold value of the distance about the interior points.

### 3.3. Classification

After filtering, we apply a perspective transformation to a standard rectangular image of fixed size for each parallelogram candidate and then use the YOLOv5 model to classify them. As different faults in the infrared images differ only in shape, we treat all hotspots as the same category. In other words, both dotted hotspots and rectangular hotspots are labeled as the same category, and they are distinguished by the bounding box of detection results. YOLOv5 is a family of object detection architectures, including YOLOv5s, YOLOv5m, YOLOv5l, and YOLOv5x, which trade off speed and accuracy. Due to the importance of real time, we choose YOLOv5s, using CSPDarknet53 with an SPP layer as the backbone, PANet as the neck, and YOLO detection head. We modify the weight ratio of classification loss to regression loss to 3:1 and manually adjust the anchor box size, and use the official settings of YOLOv5s for the rest of the settings. Define the image *I* of w×h to obtain the bounding box result $B=\left\{b_{i}=\left(x_{i},y_{i},w_{i},h_{i}\right),i=1,\ldots ,N\right\}$ by YOLOv5. To reduce the false alarm rate of small target hotspots, those bounding boxes that satisfy the condition $w_{i}<\frac{1}{m}w\;or\;h_{i}<\frac{1}{n}h$ are ignored. The above condition is determined by the size of solar panels (consisting of 10×6 battery cells). We set m=20,n=12; i.e., we will ignore the half cell size hotspot. Any image *I* will be divided into the following three categories:

1. Normal solar panels, B=∅;
2. Rectangular hotspots, $\exists b_{i}\in B,w_{i}>0.8w$;
3. Dotted hotspots, otherwise.

## 4. Experimental Results

### 4.1. Datasets and Implementation

We collect a dataset of infrared solar panel images taken by a DJI Manifold 2 UAV, including a train set (1200 images) and a test set (860 images). These images are carefully annotated by Computer Vision Annotation (CVAT, https://cvat.org). We use perspective transformation to crop out training images with a 128 × 64 pixel size and manually select 15,100 of them for training YOLOv5. The experimental settings of key components are presented below.

SEPAN is trained for nine epochs during edge detection training using a stochastic gradient descent (SGD) optimizer. A data augmentation of random flip, random rotation, and random gamma is applied to the input image (640 × 512 pixels). We set the batch size to 8, and the learning rate is set to $1\times 10^{-6}$, and divided by ten after every three epochs. At the same time, the weight decay is 0.0002, and the momentum is set to 0.9. The backbone is initialized with the pre-trained VGG16 [38] model, and other weights are initialized from a zero-mean Gaussian distribution with a standard deviation of 0.01. The training of all experiments is based on the PyTorch library [46] and is carried out on a GeForce GTX 2080Ti GPU. Since the filtering process does not require training, we only set the relevant thresholds as $N_{o}=180,t_{o}=15^{\circ }$ for the main direction filtering. We use an SGD optimizer for the training of YOLOv5 and use 0.01 as the initial learning rate with the cosine schedule. Here, the training epochs are set to 300, the batch size is 64, the mini-batch size is 16, the IoU threshold is set to 0.20, and the momentum and weight decay are 0.937 and 0.0005, respectively. We keep the same mosaic augmentation as [23,24] for 15,100 crop images. Due to solid class imbalance, we record recall, precision, and macro F1 score instead of accuracy.

In addition, we also evaluate the edge detection method on BSDS500. There are 200, 100, and 200 images in a BSDS500 training set, validation set, and test set, respectively. We use the same data augmentation as in [31,32,34,37], including flipping, scaling, and rotation to enlarge the training set 96 times. At the same time, we also add an additional flipped PASCAL VOC Context dataset to the training set. The model settings and operating environment are the same as the dataset of solar panel images. F-measures at both the Optimal Dataset Scale (ODS) and the Optimal Image Scale (OIS) are recorded for evaluation.

### 4.2. Results on Solar Panel Image Dataset

**Figure 7:**
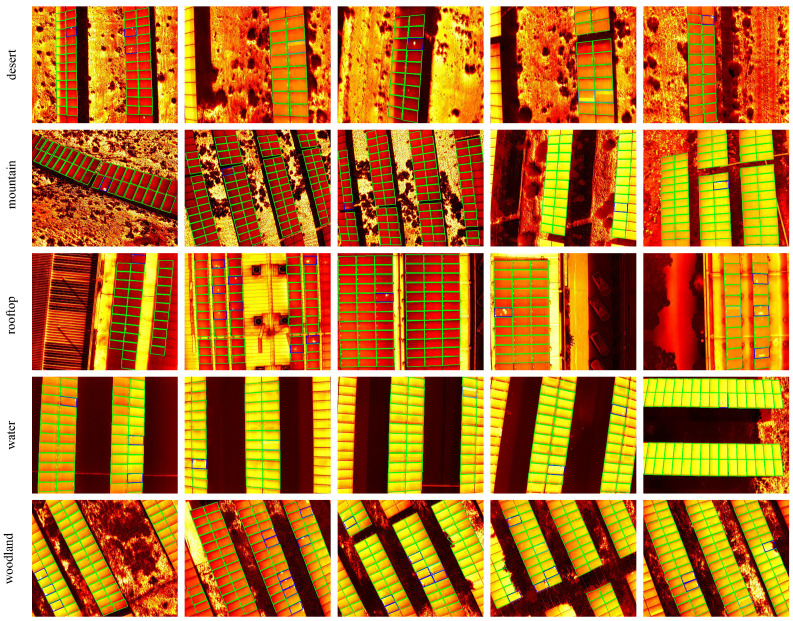
Some test results under different environments. By the way, we mark the normal, dotted, and rectangular solar panels as green, dark blue, and light blue parallelograms.

We evaluate the performance of our method on different types of PV plants. The final detection results are shown in Figure 7. Note that both dotted-faulty and rectangular-faulty solar panels compose faulty panels, which should be handled. It is true that dotted-faulty solar panels and rectangular-faulty solar panels could be mistaken. Fortunately, that mistake does not matter from a practical perspective. Therefore, dotted-faulty solar panels and rectangular-faulty solar panels can be combined into faulty ones, which are also named as macro ones. It can be seen that, except for the PV plant on water, the rest have strong background interference. We show comparison results in Table 1. It achieves a 0.9444 macro F1 score in all test data and demonstrates the strategy’s effectiveness. We can observe the following phenomena: (1) The normal has reached more than 99% of the indicators due to an imbalance class effect. The dotted faults are more difficult to detect than the rectangular ones as dotted faults are usually caused by a smaller fault area. (2) Considering the complexity of PV plants’ background area, our method can achieve better performance on the roof and the water. (3) It is crucial to find faults in practice. Therefore, our method is more inclined to a high recall rate at the cost of a little bit high false alarm rate.

To demonstrate the effectiveness of the contour filter strategy, we conduct ablation studies on the dataset of solar panels. The purpose of filtering is to remove irrelevant contours of the background after edge detection. Therefore, the accuracy can directly judge the effectiveness of filtering. As shown in Table 2, we have explored the impact of a minimal enclosing parallelogram, coarse filtering, main direction filtering, and RANSAC filtering on accuracy. Note that T/F represents the numbers of contours filtered correctly and incorrectly, respectively. We found that a coarse filter can filter out the most background contours, increasing the accuracy by about 1.5%. The main direction filtering and RANSAC filtering are designed to identify the background contours whose shape is similar to that of solar panels. Thanks to the more accurate shape representation, the minimal enclosing parallelogram also improves the accuracy by about 0.5% compared with the rotated rectangle.

### 4.3. Results on BSDS500

**Figure 8:**
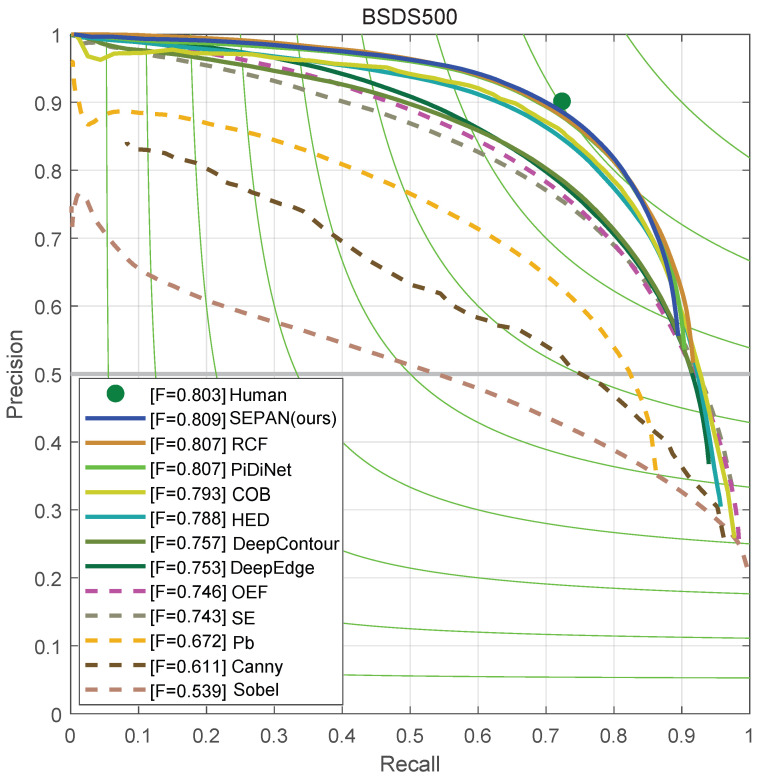
Precision/recall curves on the BSDS500 dataset.

We compare our methods with other edge detection approaches, including both traditional ones and learning-based ones, in Table 3 and Figure 8. The results show that ours reached 0.809 ODS, which outperforms most of the deep learning-based edge detectors and confirms the effectiveness of our bottom–up self-attention structure. When the baseline does not use SEPAN, the ODS of 0.805 is achieved, which still exceeds most methods, including HED [31], CED [32], and AMH-Net [33]. Note the SEPAN structure has almost no effect on the inference speed, but increases the accuracy by about 0.4%. In addition, we also provide a lightweight model, SEPAN-tiny, which only replaces the backbone network with separable convolution and achieves 128 fps. Figure 8 shows a visual comparison among several current edge detectors and SEPAN on the BSDS500 test set.

The results of top–down and bottom–up structural ablation experiments are shown in Table 4. In this experiment, we use the 200 images of the BSDS500 training set and the VOC dataset for training and record the metrics on the BSDS500 validation set. As expected, the bottom–up one is easier to obtain more precise positioning in edge detection than the top–down one, and can achieve better performance. We also add an SE Block as a comparison, producing better results. Interestingly, the SE Block brings a 0.2% improvement for the bottom–up structure, but almost nothing for the top–down one. We believe that the bottom–up structure is more suitable for the multi-scale fusion of edge detection, which is verified by the experimental results.

## 5. Conclusions

This paper presents a deep edge-based application for fault detection of solar panels. Our method, DEBFD, takes infrared images of solar panels as input and detects dotted and rectangular faults. DEBFD consists of three parts—edge detection, contour filter, and classification—which are fulfilled by the advanced deep learning networks SEPAN and YOLOv5 and a machine learning contour filter, respectively. DEBFD achieved a high macro F1 score on the 860 images taken from real scenarios. It is worth mentioning that SEPAN is our proposed bottom–up self-attention network and can effectively and accurately locate edges. Moreover, experiments on public datasets also confirm the effectiveness of our methods. In summary, DEBFD shows promising results in multiple real environments and is expected to find broad applications of PV automatic detection. Note that DEBFD may mistake dotted faults and rectangular faults of solar panels. In the future, we will explore more distinct features to distinguish dotted and rectangular faults and facilitate automatic fault classification.

## Figures and Tables

**Table 1:** Indicators of three types of solar panels in different scenarios (desert, mountain, roof, water, and woodland). Sum represents images in all scenarios. We record the precision (P), recall (R), F1 score. Note that the best ones are colored in red.

Type	Normal			Dotted			Rectangular			Macro		
	P	R	F1	P	R	F1	P	R	F1	P	R	F1
desert	0.9965	0.9941	0.9953	0.8892	0.9278	0.9081	0.9130	0.9459	0.9292	0.9330	0.9560	0.9442
mountain	0.9970	0.9956	0.9963	0.8864	0.9265	0.9060	0.9315	0.9315	0.9315	0.9384	0.9512	0.9446
roof	0.9961	0.9961	0.9961	0.9150	0.9032	0.9091	0.9387	0.9787	0.9583	0.9500	0.9594	0.9545
water	0.9980	0.9957	0.9969	0.8878	0.9340	0.9103	0.9182	0.9806	0.9484	0.9347	0.9701	0.9519
woodland	0.9950	0.9917	0.9933	0.8565	0.9052	0.8802	0.8878	0.9255	0.9062	0.9131	0.9408	0.9266
sum	0.9967	0.9948	0.9958	0.8847	0.9214	0.9027	0.9167	0.9533	0.9347	0.9328	0.9565	0.9444

**Table 2:** Ablation studies on a minimal enclosing parallelogram, coarse, main direction (MD), and RANSAC filter. T/F represents the numbers of correct and incorrect excluded contours, respectively, and we record the accuracy of the final results.

MEP	Coarse	MD	RANSAC	T/F	Acc.
✕	✕	✕	✕	0/0	0.9582
✓	✕	✕	✕	0/0	0.9634
✓	✓	✕	✕	416/3	0.9783
✓	✓	✓	✕	449/4	0.9872
✓	✓	✓	✓	474/4	0.9917

**Table 3:** Comparison with other methods on the BSDS500 dataset. ‡ indicates the speed with our implementations based on an NVIDIA RTX 2080 Ti GPU. † means the cited GPU speed.

Method	ODS	OIS	FPS
Human	0.803	0.803	
Canny [27]	0.611	0.676	28
Pb [30]	0.672	0.695	-
SE [28]	0.743	0.763	12.5
OEF [29]	0.746	0.77	2/3
HED [31]	0.788	0.808	$30^{\;†}$
CED [32]	0.794	0.811	-
AMH-Net [33]	0.798	0.829	-
RCF [34]	0.806	0.823	$30^{\;†}$
LPCB [35]	0.808	0.824	$30^{\;†}$
BDCN [36]	0.820	0.838	$47^{\;‡}$
PiDiNet [37]	0.807	0.823	92
Baseline	0.805	0.821	$30^{\;‡}$
SEPAN (ours)	0.809	0.827	$30^{\;‡}$
SEPAN-Tiny (ours)	0.789	0.804	$128^{\;‡}$

**Table 4:** Ablation on top–down and bottom–up structure. The training data are the BSDS500 training set and VOC dataset, and the evaluation data are the BSDS500 validation set.

	ODS	OIS
baseline	0.774	0.789
top–down	0.776	0.792
SE+top–down	0.776	0.793
bottom–up	0.777	0.796
SE+bottom–up	0.779	0.797

## Data Availability

Data are contained within the article.

## References

[1] Ram M., Bogdanov D., Aghahosseini A., Gulagi A., Oyewo A.S., Child M., Caldera U., Sadovskaia K., Farfan J., Barbosa L.S.N.S. (2019). Global Energy System Based on 100% Renewable Energy—Power, Heat, Transport and Desalination Sectors.

[2] Venkatesh S N., Sugumaran V. (2020). Fault diagnosis of visual faults in photovoltaic modules: A Review. Int. J. Green Energy.

[3] Madeti S.R., Singh S.N. (2017). A comprehensive study on different types of faults and detection techniques for solar photovoltaic system. Sol. Energy.

[4] Bhaskaranand M., Gibson J.D. Low-complexity video encoding for UAV reconnaissance and surveillance. Proceedings of the 2011-MILCOM 2011 Military Communications Conference.

[5] Barbedo J.G.A., Koenigkan L.V., Santos T.T., Santos P.M. (2019). A study on the detection of cattle in UAV images using deep learning. Sensors.

[6] Sa I., Hrabar S., Corke P. (2015). Outdoor flight testing of a pole inspection UAV incorporating high-speed vision. Field and Service Robotics.

[7] Su B., Chen H., Liu K., Liu W. (2021). RCAG-Net: Residual Channelwise Attention Gate Network for Hot Spot Defect Detection of Photovoltaic Farms. IEEE Trans. Instrum. Meas..

[8] Akram M.W., Li G., Jin Y., Chen X., Zhu C., Ahmad A. (2020). Automatic detection of photovoltaic module defects in infrared images with isolated and develop-model transfer deep learning. Sol. Energy.

[9] Dotenco S., Dalsass M., Winkler L., Würzner T., Brabec C., Maier A., Gallwitz F. Automatic detection and analysis of photovoltaic modules in aerial infrared imagery. Proceedings of the 2016 IEEE Winter Conference on Applications of Computer Vision (WACV).

[10] Vega Díaz J.J., Vlaminck M., Lefkaditis D., Orjuela Vargas S.A., Luong H. (2020). Solar panel detection within complex backgrounds using thermal images acquired by UAVs. Sensors.

[11] Chen J., Li Y., Ling Q. Hot-Spot Detection for Thermographic Images of Solar Panels. Proceedings of the 2020 Chinese Control and Decision Conference (CCDC).

[12] Rahman M.R.U., Chen H. (2020). Defects inspection in polycrystalline solar cells electroluminescence images using deep learning. IEEE Access.

[13] Ge C., Liu Z., Fang L., Ling H., Zhang A., Yin C. (2020). A hybrid fuzzy convolutional neural network based mechanism for photovoltaic cell defect detection with electroluminescence images. IEEE Trans. Parallel Distrib. Syst..

[14] Lin H.H., Dandage H.K., Lin K.M., Lin Y.T., Chen Y.J. (2021). Efficient cell segmentation from electroluminescent images of single-crystalline silicon photovoltaic modules and cell-based defect identification using deep learning with pseudo-colorization. Sensors.

[15] Deitsch S., Christlein V., Berger S., Buerhop-Lutz C., Maier A., Gallwitz F., Riess C. (2019). Automatic classification of defective photovoltaic module cells in electroluminescence images. Sol. Energy.

[16] Su B., Chen H., Chen P., Bian G., Liu K., Liu W. (2020). Deep learning-based solar-cell manufacturing defect detection with complementary attention network. IEEE Trans. Ind. Inform..

[17] Li X., Li W., Yang Q., Yan W., Zomaya A.Y. (2020). Edge-Computing-Enabled Unmanned Module Defect Detection and Diagnosis System for Large-Scale Photovoltaic Plants. IEEE Internet Things J..

[18] Mehta S., Azad A.P., Chemmengath S.A., Raykar V., Kalyanaraman S. Deepsolareye: Power loss prediction and weakly supervised soiling localization via fully convolutional networks for solar panels. Proceedings of the 2018 IEEE Winter Conference on Applications of Computer Vision (WACV).

[19] Ren S., He K., Girshick R., Sun J. (2015). Faster r-cnn: Towards real-time object detection with region proposal networks. Adv. Neural Inf. Process. Syst..

[20] Redmon J., Divvala S., Girshick R., Farhadi A. You only look once: Unified, real-time object detection. Proceedings of the IEEE Conference on Computer Vision and Pattern Recognition.

[21] Redmon J., Farhadi A. YOLO9000: Better, faster, stronger. Proceedings of the IEEE Conference on Computer Vision and Pattern Recognition.

[22] Redmon J., Farhadi A. (2018). Yolov3: An incremental improvement. arXiv.

[23] Bochkovskiy A., Wang C.Y., Liao H.Y.M. (2020). Yolov4: Optimal speed and accuracy of object detection. arXiv.

[24] Jocher G. (2021). YOLOv5. https://github.com/ultralytics/yolov5.

[25] Arbelaez P., Maire M., Fowlkes C., Malik J. (2010). Contour detection and hierarchical image segmentation. IEEE Trans. Pattern Anal. Mach. Intell..

[26] Prewitt J.M.S. (1970). Object enhancement and extraction. Picture Processing and Psychopictorics.

[27] Canny J. (1986). A computational approach to edge detection. IEEE Trans. Pattern Anal. Mach. Intell..

[28] Dollár P., Zitnick C.L. (2014). Fast edge detection using structured forests. IEEE Trans. Pattern Anal. Mach. Intell..

[29] Hallman S., Fowlkes C.C. Oriented edge forests for boundary detection. Proceedings of the IEEE Conference on Computer Vision and Pattern Recognition.

[30] Martin D.R., Fowlkes C.C., Malik J. (2004). Learning to detect natural image boundaries using local brightness, color, and texture cues. IEEE Trans. Pattern Anal. Mach. Intell..

[31] Xie S., Tu Z. Holistically-nested edge detection. Proceedings of the IEEE International Conference on Computer Vision.

[32] Wang Y., Zhao X., Huang K. Deep crisp boundaries. Proceedings of the IEEE Conference on Computer Vision and Pattern Recognition.

[33] Xu D., Ouyang W., Alameda-Pineda X., Ricci E., Wang X., Sebe N. (2018). Learning deep structured multi-scale features using attention-gated crfs for contour prediction. arXiv.

[34] Liu Y., Cheng M.M., Hu X., Wang K., Bai X. Richer convolutional features for edge detection. Proceedings of the IEEE Conference on Computer Vision and Pattern Recognition.

[35] Deng R., Shen C., Liu S., Wang H., Liu X. Learning to predict crisp boundaries. Proceedings of the European Conference on Computer Vision (ECCV).

[36] He J., Zhang S., Yang M., Shan Y., Huang T. Bi-directional cascade network for perceptual edge detection. Proceedings of the IEEE/CVF Conference on Computer Vision and Pattern Recognition.

[37] Su Z., Liu W., Yu Z., Hu D., Liao Q., Tian Q., Pietikäinen M., Liu L. Pixel difference networks for efficient edge detection. Proceedings of the IEEE/CVF International Conference on Computer Vision.

[38] Simonyan K., Zisserman A. (2014). Very deep convolutional networks for large-scale image recognition. arXiv.

[39] Lin T.Y., Dollár P., Girshick R., He K., Hariharan B., Belongie S. Feature pyramid networks for object detection. Proceedings of the IEEE Conference on Computer Vision and Pattern Recognition.

[40] Liu S., Qi L., Qin H., Shi J., Jia J. Path aggregation network for instance segmentation. Proceedings of the IEEE Conference on Computer Vision and Pattern Recognition.

[41] Ghiasi G., Lin T.Y., Le Q.V. Nas-fpn: Learning scalable feature pyramid architecture for object detection. Proceedings of the IEEE/CVF Conference on Computer Vision and Pattern Recognition.

[42] Tan M., Pang R., Le Q.V. Efficientdet: Scalable and efficient object detection. Proceedings of the IEEE/CVF Conference on Computer Vision and Pattern Recognition.

[43] Hu J., Shen L., Sun G. Squeeze-and-excitation networks. Proceedings of the IEEE Conference on Computer Vision and Pattern Recognition.

[44] Suzuki S. (1985). Topological structural analysis of digitized binary images by border following. Comput. Vision Graph. Image Process..

[45] Schwarz J., Teich J., Welzl E., Evans B. (1994). On Finding a Minimal Enclosing Parallelogram.

[46] Paszke A., Gross S., Massa F., Lerer A., Bradbury J., Chanan G., Killeen T., Lin Z., Gimelshein N., Antiga L. (2019). Pytorch: An imperative style, high-performance deep learning library. Adv. Neural Inf. Process. Syst..

